# A Paradoxical Effect of Interleukin-32 Isoforms on Cancer

**DOI:** 10.3389/fimmu.2022.837590

**Published:** 2022-02-25

**Authors:** Saerok Shim, Siyoung Lee, Yasmin Hisham, Sinae Kim, Tam T. Nguyen, Afeisha S. Taitt, Jihyeong Hwang, Hyunjhung Jhun, Ho-Young Park, Youngmin Lee, Su Cheong Yeom, Sang-Yeob Kim, Yong-Gil Kim, Soohyun Kim

**Affiliations:** ^1^Laboratory of Cytokine Immunology, Department of Biomedical Science and Technology, Konkuk University, Seoul, South Korea; ^2^YbdYbiotech Research Center, Seoul, South Korea; ^3^Technical Assistance Center, Korea Food Research Institute, Wanju, South Korea; ^4^Research Group of Functional Food Materials, Korea Food Research Institute, Wanju, South Korea; ^5^Department of Medicine, Pusan Paik Hospital, Collage of Medicine, Inje University, Busan, South Korea; ^6^Graduate School of International Agricultural Technology, Seoul National University, Pyeongchang, South Korea; ^7^Convergence Medicine Research Center, Asan Institute for Life Science, Asan Medical Center, Seoul, South Korea; ^8^Division of Rheumatology, Department of Internal Medicine, Asan Medical Center, University of Ulsan College of Medicine, Seoul, South Korea; ^9^College of Veterinary Medicine, Konkuk University, Seoul, South Korea

**Keywords:** interleukin-32, tumor microenvironment, stromal tumor, hypoxia, metastasis

## Abstract

IL-32 plays a contradictory role such as tumor proliferation or suppressor in cancer development depending on the cancer type. In most cancers, it was found that the high expression of IL-32 was associated with more proliferative and progression of cancer. However, studying the isoforms of IL-32 cytokine has placed its paradoxical role into a wide range of functions based on its dominant isoform and surrounding environment. IL-32β, for example, was found mostly in different types of cancer and associated with cancer expansion. This observation is legitimate since cancer exhibits some hypoxic environment and IL-32β was known to be induced under hypoxic conditions. However, IL-32θ interacts directly with protein kinase C-δ reducing NF-κB and STAT3 levels to inhibit epithelial-mesenchymal transition (EMT). This effect could explain the different functions of IL-32 isoforms in cancer. However, pro- or antitumor activity which is dependant on obesity, gender, and age as it relates to IL-32 has yet to be studied. Obesity-related IL-32 regulation indicated the role of IL-32 in cancer metabolism and inflammation. IL-32-specific direction in cancer therapy is difficult to conclude. In this review, we address that the paradoxical effect of IL-32 on cancer is attributed to the dominant isoform, cancer type, tumor microenvironment, and genetic background. IL-32 seems to have a contradictory role in cancer. However, investigating multiple IL-32 isoforms could explain this doubt and bring us closer to using them in therapy.

## Introduction

The human interleukin-32 (IL-32) is a novel cytokine that exerts both pro and anti-inflammatory roles. IL-32 gene is found in higher primates, and it is located in chromosome 16 at p13.3 encoding for various isoforms. IL-32 plays an essential role in innate and adaptive immune responses, and it induces various cytokines such as tumor necrosis factor (TNF)-α, IL-1β, IL-6, and IL-8 (1). After its identification, it has been studied in inflammatory disorders including autoimmune diseases and cancers (2, 3).

In cancer, inflammatory tumor microenvironment such as cytokines, IL-32 plays a crucial role in its progression (4). Therefore, IL-32 has been studied for its tumor control direction in several cancer types. However, paradoxical effects have been reported regarding IL-32 on cancers, which may be attributed to the dominant isoform, cancer type, and genetic background. On the one hand, IL-32 was reported to augment cancer progression, proliferation, invasion, and metastasis in many tumors including acute myeloid leukemia (AML), hepatocellular carcinoma (HCC), and breast, lung, colon, pancreatic, and gastric cancers (5–12). On the other hand, it was also reported to have anticancer activity in different cancers including acute and chronic myeloid leukemia (AML and CML) and breast, lung, and colon cancers (13–19).

IL-32 gene was found to have several isoforms based on different alternative splicing sites. It has eight exons in which the first exon does not translate into amino acids. Mainly, seven isoforms were depicted and were identified separately which are IL-32α, IL-32β, IL-32γ, IL-32δ, IL-32ε, IL-32ζ, and IL-32θ (3). IL-32α, IL-32β, IL-32γ, and IL-32δ were primarily detected in IL-2-stimulated human NK cells. While IL-32ε and IL-32ζ were observed to be expressed in the activated T cells (20), and IL-32θ was found within dendritic, Jurkat, human leukemia T cells (21). Structural characteristics of the seven IL-32 isoforms were reviewed based on the IL-32 eleven protein domains (3). However, a lot of knowledge is waiting to be revealed regarding IL-32 isoforms, such as their specific receptors. These isoforms displayed distinctive roles and consequences in different conditions although they are deficient in signal peptides. Therefore, a functional comparison between these isoforms as well as specific antibodies to detect IL-32 isoforms is considered necessary.

Nevertheless, what has been discovered so far still lacks explicit knowledge about IL-32 function in cancers. It is known that many factors can affect the disease outcome, especially in cancer, yet this much contradiction was not reported to any cytokine other than IL-32. This contradiction is mainly due to not considering IL-32 isoforms in most of the studies. In this review, we aim to analyze previous reports to address the most probable functions of IL-32 on different cancers to provide recommendations for further studies and unravel possible therapeutic options.

## IL-32 in Cancer Proliferation and Apoptosis

IL-32 was found to play two contradictory roles in cancer development among various cancer types, one role as a critical proliferation and growth factor and the other as a tumor suppressor. Higher expression of IL-32 was found to be associated with more proliferative and progression in the following cancers, AML, cutaneous T-cell lymphoma (CTCL), gastric B-cell lymphoma (GBCL), multiple myeloma (MM), HCC, and breast, lung, colon, pancreatic, gastric, and esophageal cancers (5–12, 22–25).

**Figure 1:**
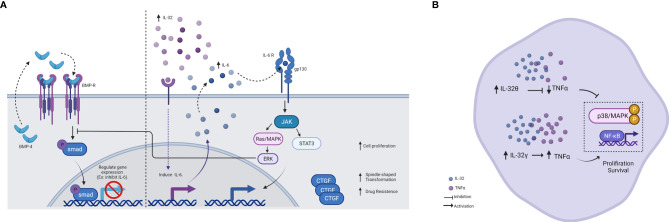
Schematic illustration showing the effect of IL-32. **(A)** Schematic illustration showing the effect of IL-32 on the BMP pathway and IL-6 induction. In the presence of BMP4, it feeds the loop and binds to the BMP receptor, activating SMAD and thus regulating gene expression. IL-6 is inhibited by this regulation. On the other hand, in the presence of IL-32, IL-6 is induced and activates several pathways. One of these pathways is ERK, which in turn inhibits SMAD. Therefore, IL-6 induced by IL-32 acts as negative feedback for the BMP pathway, as results of cell proliferation increased. IL-32 was found to increase the expression (either directly or indirectly) of the connective tissue growth factor (cTGF), as results of spindle-shape transformation increased, and thus invasion and metastasis occurred. **(B)** Schematic illustration showing the different effects of IL-32 isoform in AML. IL-32γ was shown to induce TNF-α production and activate NF-κB and MAPK signaling pathways and therefore, increased proliferation and survival. Whereas, IL-32θ was shown to inhibit TNF-α and phosphorylated p38 MAPK and NF-κB, thus, reducing cancer progression. This makes IL-32θ to be considered a potent inhibitor of TNF-α in patients with AML. Figure created by BioRender App.

In acute leukemia peripheral blood of patients, IL-32 was closely related to the disease development (5). Recently, AML-derived mesenchymal stem cells (AML-MSCs) when cocultured with K562/K562 ADM cells, showed changes in the expression of IL-6 and IL-32 cytokines. These data suggested its effect on proliferation, invasion, metastatic, and drug resistance through dysregulation of bone morphogenetic protein-4 (BMP4) pathway as well as increased the connective tissue growth factor (cTGF) in K562 ADM cells (**Figure 1A**) (6). BMP pathways modulate the expression of target genes, and it was found to inhibit the expression of IL-6, suggesting a similar effect on IL-32 (26, 27). Therefore, dysregulation of BMP4 seems to have the opposite effect and thus increase the expression of cytokines. Moreover, a recent study has revealed a cancer suppressor effect when the BMP4 signaling pathway is activated (28). On the other hand, cTGF promotes the spindle shape transformation that is responsible for the invasion and metastatic thus, contributing to the disease progress.

Although studies mentioned above indicated the enhancement role of IL-32 in AML survival, an inhibitory effect of this cytokine was also reported, specifically IL-32θ isoform, by regulating TNF-α production in AML (13). In this study, they divided AML patients into two groups based on the presence of IL-32θ and found that IL-32θ inhibits the increment of TNF-α. They then confirm that IL-32θ inhibited phosphorylation of p38 mitogen-activated protein kinase (MAPK) and nuclear factor-κB (NF-κB) *in vivo*. In addition, IL-32θ attenuated TNF-α promoter activity and the binding of NF-κB with the TNF-α promoter (**Figure 1B**). Moreover, another inhibitory effect of IL-32 was reported in CML cells through enhancing natural killer (NK) cell-mediated killing (14). Here, the NK killing activity is achieved through stimulation of both the Fas receptor and UL16-binding protein (ULBP), ligands of NKG2D in NK. The performance of more IL-32 experiments in the absence of specific IL-32 isoform characterization may show vast contradictions. The wide range of activities can be confusing at this moment, but studying its isoforms in depth may shed light on this seemingly paradoxical function.

**Figure 2:**
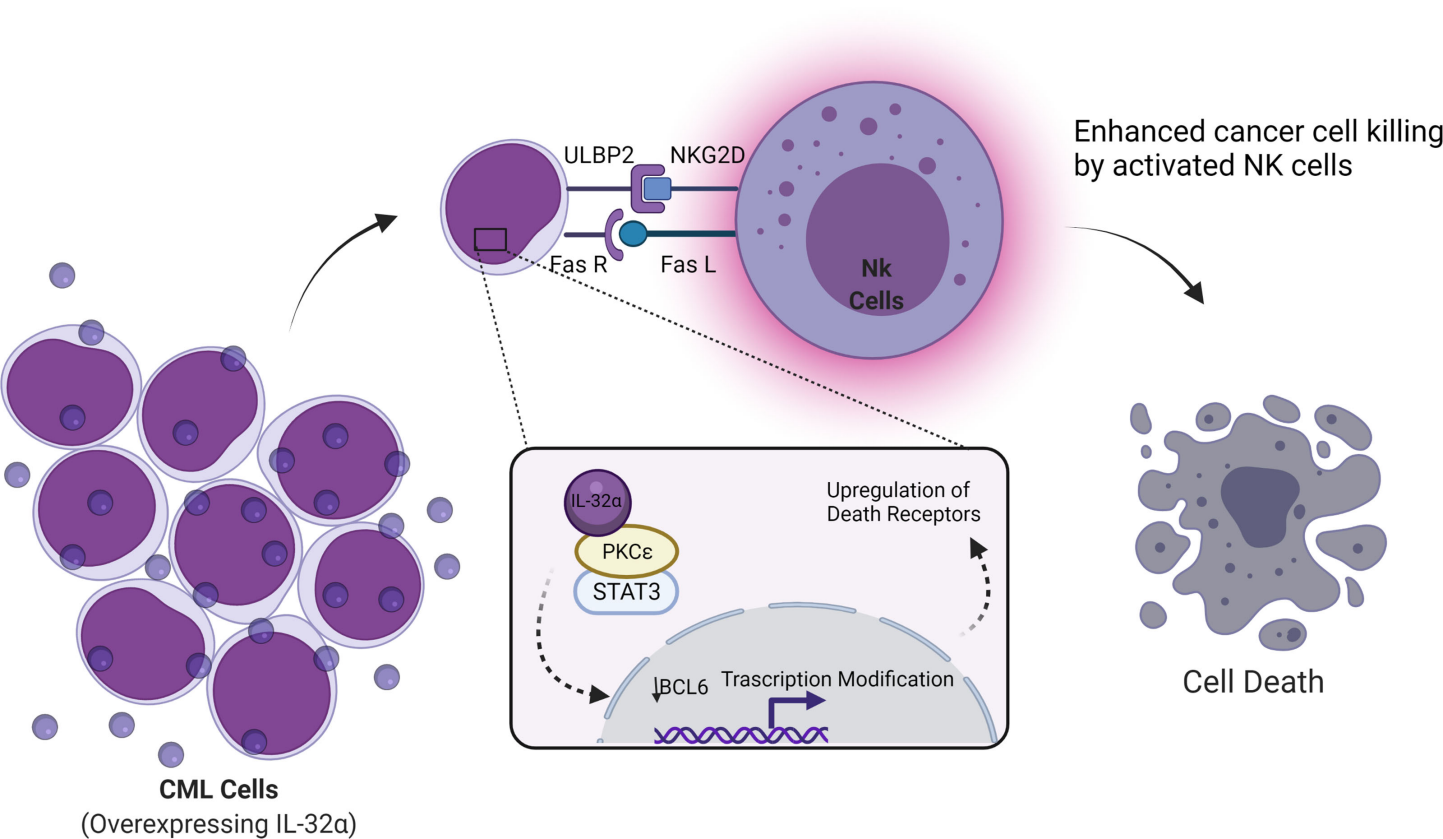
Schematic illustration showing cancer cell death by IL-32α in CML and lymphoma. Cancer cell death was reported when the IL-32α isoform is expressed in CML or lymphoma. This cancer inhibitory effect occurs through enhancing natural killer (NK) cell-mediated killing. PKCℇ inhibits apoptosis and the promotion of cell survival, by regulating several pathways, one of PKCℇ regulations is the activation of STAT3. IL-32α binds to PKCℇ and inhibits its functions and regulations. As a result, transcriptional modifications occurred including the downregulation of Bcl-6 and the upregulation of death receptors (ULBP2 and Fas receptor) resulting in NK cell-mediated killing by the stimulation of both Fas receptor and ULBP. ULBP is a ligand of NKG2D in NK. Figure created by BioRender App.

IL-32α induces this stimulation through activation of p38 MAPK. IL-32α also inhibits B-cell CLL lymphoma through regulation on epigenetic posttranslational modifications. B-cell lymphoma-6 (Bcl-6) has been associated with progression of lymphomas and is considered a master regulator of cellular processes (29). Bcl-6 was found to be inhibited by IL-32α *via* the production of IL-6 and PKCϵ-mediated cell adhesion (30). PKCϵ is known to have two major roles that are inhibition of apoptosis and promotion of cell survival as one of its regulated pathways in the activation of STAT3 (31, 32). IL-32 regulates this activation and induces apoptosis (**Figure 2**).

Active PKCϵ crosstalks to multiple signal transduction pathways result in the following two major cellular effects: (1) inhibition of apoptosis and (2) promotion of cell survival. PKCϵ-regulated cell survival pathways include Stat3 activation, expression of growth-stimulating cytokines (TNF-α, GM-CSF, and G-CSF), and growth factors (e.g., EGFR). PKCϵ mediates inhibition of apoptosis *via* inhibition of FADD expression. All these pathways in fact constitute a network.

## The Effect of Cytokine Signal Pathways in the Role of IL-32 in Cancer

The influence of IL-32 in tumor growth through the inactivation of NF-κB and signal transducer and activator of transcription 3 (STAT3) pathways have been mentioned earlier (33). Moreover, IL-32γ downregulates vital cancer progression proteins including antiapoptotic, cell proliferation, and tumor-promoting genes, while upregulating the apoptotic genes. On the contrary, IL-32γ isoform is shown to diminish the levels of cytokines that promote tumor growth such as TNF-α, IL-1β, and IL-6, whereas the levels of IL-10 cytokine, a tumor growth-inhibiting cytokine, were elevated. The anticancer activity of IL-32γ was found in several cancer cells but not in melanomas like colon, prostate, liver, and lung. It also induces the activation of cytotoxic T cells and NK cells to the tumor site to expand the cancer eradication effect (33, 34) as well as recently, it showed better immunotherapy response (35).

Later, IL-32β has been found to play an antitumor activity role as it downregulates vital cancer progression proteins including antiapoptotic proteins, proliferation, and cell growth regulatory proteins through the same pathways, NF-κB and STAT3. In addition, IL-32β was found to induce the expressions of proapoptotic proteins and regulate the release of cytokines in colon and prostate cancer cells (15). Nevertheless, higher expression of IL-32α has been found to activate NF-κB and STAT3 pathways and induce the production of IL-6, thus supporting the cancer proliferation and progression in MM patients (25). Therefore, finding the exact function of the IL-32 isoform is still a sensitive consideration and may be influenced not only by its isoform but also with cancer type as well as the whole tumor microenvironment.

We have mentioned the anticancer activity of IL-32γ in colon cancers, which is considered through activation of p38 MAPK pathways (16). Moreover, IL-32α and IL-32θ have been found to suppress the effect on colon cancer, as well (17, 18, 36). In the case of the expression IL-32α, the expression of TNF receptor 1 and the production of reactive oxygen species was increased, thus facilitating apoptosis and prolonged JNK activation. At the same time, several studies have mentioned the contradictory role of IL-32 in colon cancer (7, 37, 38) whereas IL-32 was found to be upregulated and associated with poor survival. In this regard, it is worth mentioning a finding that provides evidence on the contribution of IL-32α in the development of obesity-associated colon cancer by favorably remodeling cytokine for tumor growth (39). According to the currently available data, we can suppose that in colon cancer, IL-32α has both pro- and antitumor activity depending on other factors such as obesity, gender, and/or age-related factors which have not been studied yet. However, obesity-related IL-32 manipulation indicates that IL-32 could play a role in cancer metabolism as well as inflammation.

## IL-32 in Breast Cancer

In breast cancer, its metabolism regulation was found to be influenced by IL-32β expression. IL-32β was stimulated due to hypoxia and found to increase glycolysis and Src (proto-oncogene tyrosine-protein kinase) activation by activating lactate dehydrogenase and inhibiting Src dephosphorylation, respectively (40). This metabolic change is achieved through lactate dehydrogenase activation when IL-32β translocated into mitochondria due to its accumulation. Moreover, the inhibition of hypoxia-induced IL-32β impairs tumor cell growth, making it a potential drug target (8, 40). Interestingly, when both mRNA and protein levels were evaluated, IL-32 demonstrated isoform switching and self-regulation, as at mRNA levels IL-32β and IL-32γ were detected. However, at the protein level, through Western blot, only IL-32β was detected (41). Another study has also reported that elevated IL-32 promoted growth, stemness, and progression in breast cancer (42). In addition, because IL-32 was found to be highly expressed in cancer tissue of triple-negative breast cancer patients, it was suggested as a probable therapeutic target (9).

**Figure 3:**
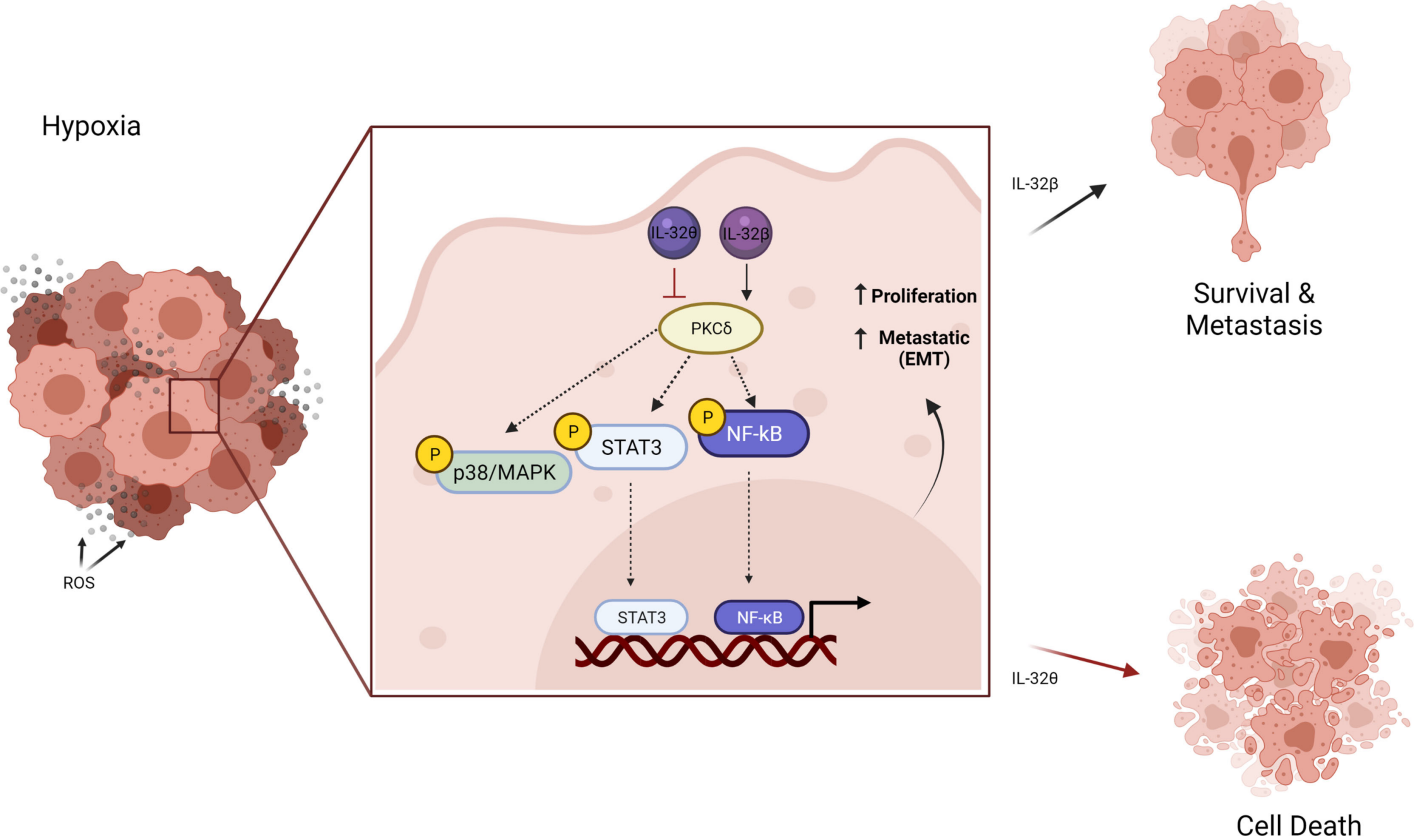
Schematic illustration showing the different effects of IL-32 isoform in cancer cells under hypoxic conditions. The elevated IL-32β interacts with PKCδ in tumor-promoting antiapoptotic signaling that increased cancer progression. On the contrary, IL-32θ interacts with PKCδ inhibiting its antiapoptotic effect and reduces NF-κB and STAT3, thus inhibiting epithelial-mesenchymal transition (EMT). Figure created by BioRender App.

Moreover, the elevation of IL-32β expression under hypoxic conditions was also found in ovarian cancer cells by reducing its degradation. They found that IL-32β interacts with protein kinase Cδ (PKCδ) thus promoting antiapoptotic function under oxidative stress, which is almost the case in breast cancer. However, more recently, IL-32θ isoform was found to utilize antiproliferative effects in breast cancer cells and initiate senescence (43, 44). Intriguingly, it was revealed that IL-32θ interacts directly with PKCδ and subsequently reduces NF-κB and STAT3 levels and thus inhibits epithelial-mesenchymal transition (EMT). This effect could provide a clue regarding the different functions of IL-32 reported in cancer. Although PKCδ is known for its proapoptotic function in cancer cells (45), it seems that PKCδ when interacting with a different isoform of IL-32 exhibits different signal therefore different effect (**Figure 3**).

Also, when IL-32 has been reviewed, the difference between these two isoforms (IL-32β and IL-32θ) was revealed to be only one motif consisting of 20 amino acids (DDFKEGHLETVAAYYEEQHP) (3). In another word, both isoforms shared the binding site for PKC, but this motif is mainly responsible for its furthered role. Therefore, it can be suggested that this motif found within isoform β but not θ (DDFKEGHLETVAAYYEEQHP) can activate PKC function and therefore enhance cancer progression. Extended physical interaction and functional studies are required to prove this conclusion. IL-32 altered the same pathway among several types of cancer; when the isoform is changed, the final effect is also changed. Therefore, it is very crucial to introduce some regulations when studying this IL-32 cytokine. It is necessary to detect the isoforms and their levels in the same study case. Isoforms should be determined in both mRNA and protein levels and recognize their specific cellular localization such as cytoplasm, extracellular, or nucleus.

## IL-32 in GI, Esophageal, Gastric, Liver, and Pancreatic Cancers

Most GI cancers include esophageal, gastric, liver (e.g., HCC), and pancreatic cancers, were found to express higher levels of IL-32, and mostly exhibit a facilitating cancer progression role. IL-32 was highly expressed in tissue and serum of patients with HCC and was associated with disease progression (46–48). The only isoform studied in this cancer type was IL-32α, and its expression was correlated with antiapoptotic signals, mainly Bcl-2 regulator protein, p38-MAPK, and NF-κB pathways. Moreover, similar activity for IL-32 in promoting cancer growth and survival was reported in pancreatic cancer (11, 49). Furthermore, its induction was facilitated through phosphatidylinositol 3−kinase/protein kinase B (PI3K/Akt) pathway-dependent NF-κB/AP-1 activation.

As IL-32 is highly expressed in serum and tissues of GI cancers, it was found with 99.5% accuracy in detected gastroesophageal cancers as a biomarker (12, 22, 37, 50–57). In both cancers, gastric and esophagus, IL-32 upregulation was coupregulated with proinflammatory cytokines such as TNF-α, IL-1β, and IL-6, suggesting its induction *via* NF-κB and STAT3 signaling pathways was linked to poor-prognosis cases. It was found that IL-32β was the dominant isoform expressed in gastric tissues with 90%, and the remaining 10% was IL-32ϵ with no detection for any other isoforms. However, their total sample was only 20, which signifies the need for further investigation into a wider cohort. The recent publication evaluated the expression of IL-32 in different immune cells from esophageal squamous cell carcinoma (ESCC) by single-cell RNA sequencing found that IL-32 may have a paradoxical effect (22). They found that IL-32 stimulates the expression of IFN-γ in CD8^+^ T cells which is responsible for the antitumor role, while in CD4^+^ T cells it induces Foxp3 expression, which accounts for the suppressor role.

## IL-32 in Cancer Angiogenesis, Invasion, and Metastasis

**Figure 4:**
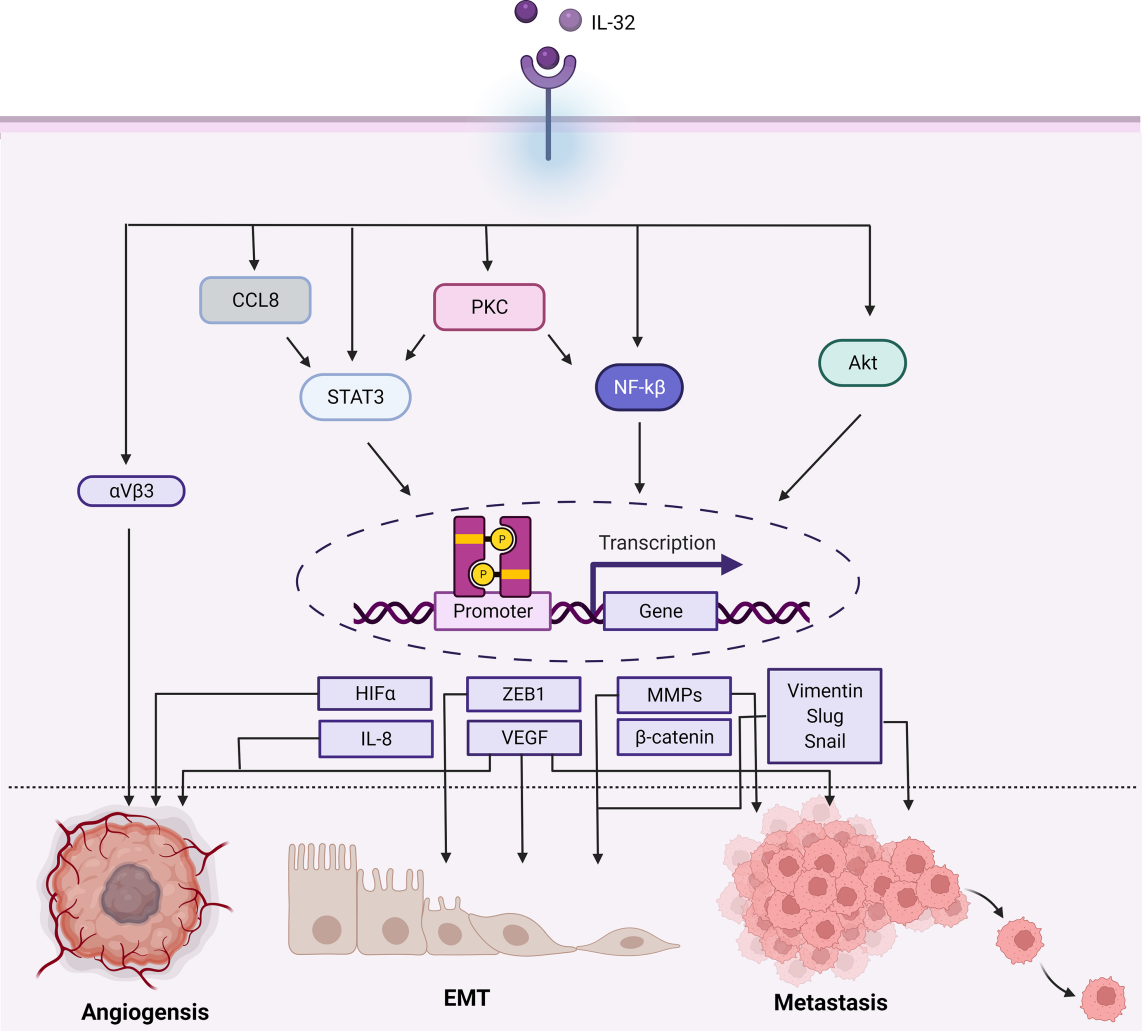
Schematic illustration showing the range of signaling pathways that are activated by IL-32 and promoting cancer progression. In terms of angiogenesis, EMT, and metastasis. In brief, IL-32 promotes the Akt, NF-kB, STAT3 (which can be activated by PKC/CCL8), and integrin αVβ3 signaling cascades, each having different transcription modifications. Therefore, regulating the activity of several transcription factors play a role in cancer such as angiogenesis, EMT, and metastasis as well as αVβ3, VEGF, and HIF-α enhance angiogenesis. The ZEB1 or B-catenin enhances EMT. VEGF is also associated with EMT and metastasis. Additionally, the transcription of MMPs (like MMP2 and MMP9), Vimentin, Slug, and Snail promotes metastasis. Figure created by BioRender App.

IL-32 numerous roles in angiogenesis, EMT, and metastasis are summarized in **Figure 4**. Angiogenesis invasion and metastatic both are features established in more aggressive tumors. Therefore, IL-32 involvement in these two processes was evaluated in several studies. Angiogenesis occurs as a response due to diminishing oxygen and nutrients, the new vessels formed provide a crucial pathway for metastasis. IL-32 was found to influence angiogenesis in glioblastoma, yet the underlying mechanism remains to be defined (58). In this study, it was found that IL-32 controls angiogenesis through integrin αVβ3, that usually expressed in new vessels and is considered the most important integrin for angiogenesis (59, 60). The expression of IL-32 was significantly increased and colocalized with integrin αVβ3. Vascular endothelial growth factor (VEGF) is a well-known critical factor for metastatic and angiogenesis and is the most expressed in advanced cancers (61). The tube formation was found to be increased in a dose-dependent manner as well. Besides, αVβ3 inhibitor reduced IL-32, and induced IL-8 (one of the advocates of angiogenesis), therefore blocking the angiogenetic effect.

Interestingly, they found that the reduction of IL-32 affects IL-8, nitric oxide, and matrix metalloproteinases 9 (MMP9), whereas levels of VEGF and TGFβ were not affected. Thus, it was concluded that the angiogenetic activity conducted by IL-32, specifically IL-32γ, was not mediated by VEGF. Since IL-32 induced IL-8, which could be the indirect way of promoting angiogenesis. IL-8 plays a role in invasion, metastasis, and angiogenesis (62). VEGF expression was found to be correlated with the expression of IL-32 in cancers with invasion and migration ability such as lung, breast, and gastric cancers although it was indicated that IL-32γ pro angiogenetic activity was not mediated by VEGF (8, 10, 50).

Matrix metalloproteinases family (MMPs) of endopeptidases having proteolytic activity play a critical role in the invasion and metastasis of tumors through their function of extracellular matrix degradation (63–65). In gastric and lung cancer, not only VEGF and IL-8 were found to be coexpressed with IL-32 but also MMP2 and MMP9 (10, 50). IL-32 significantly increased in metastatic patients of both cancer types (10, 51, 66). As mentioned above, IL-32 was highly associated with gastric cancer progression mainly due to its stimulation of cell elongation and in turn enhanced invasion and migration. This effect occurs through activation of AKT, β-catenin, and hypoxia-inducible factor 1α (HIF1- α) signaling pathways.

It was noted that the expressed IL-32 isoforms were α, β, and γ in gastric cancer samples, while the dominant isoform was IL-32β. Since IL-32γ was found to be spliced into IL-32 α and β, they evaluate the effect of IL-32γ on the gastric carcinoma cell line (TSGH9201). As a result, they found that cells overexpressing IL-32 show elongated spindle-like morphology compared to the control cells (50). Invasion stimulation in cancer cells *via* the Akt pathway was also reported within osteosarcoma cells mediated by the expression and secretion of MMP13 (67). On the other hand, in lung cancer cells MMP 2 and 9 were also found to be induced by IL-32 but *via* NF-kB (10).

The overexpression of IL-32 was found to be correlated with metastasis in ESCC and colorectal cancer (37, 38). However, one study revealed that IL-32 isoform could play an opposite migratory role in colon cancer cells (18). It was found that isoform IL-32θ represses the invasion and migration of colon cancer cells by preventing EMT. This was achieved by the interaction of IL-32θ with STAT3 to suppress ZEB1 and Bmi1 transcription which in turn avoids stemness and EMT.

Moreover, this inhibitory effect of IL-32θ was addressed in breast cancer as well, as it suppresses the binding of CCL18, a chemotactic cytokine involved in the several cancer pathogenesis and progression and associated with poor prognosis (68–70), to its receptor and therefore inhibited the further cascade of activation/phosphorylation of STAT3 (44). Phosphorylation of STAT3, regardless of its upstream activation, leads to dimerization and translocation into the nucleus. Following that, STAT3 binds to its target gene promoters and regulates their expression (71–73). MMPs are among its target genes, which in this way STAT3 is involved in regulating cancer cell migration (74, 75). In addition, STAT3 regulates VEGF and HIF1-α that are well known for their role in angiogenesis (76–78).

Taken together, STAT3 signaling pathways play a key role in cancer metastasis (73) and are found to be regulated by IL-32. The upregulation of MMPs (MMP2, MMP9, and MMP13) was also reported in cancers overexpressing IL-32 along with other EMT markers including vimentin, Slug, Snail, and ZEB1, as well as they are well known for their contribution to cancer metastatic.

## IL-32 in the Tumor Microenvironment and Stromal Tumor

The tumor microenvironment refers to the surrounding ecosystem that includes extracellular matrix, blood vessels, and an array of cells such as fibroblasts, immune cells, and heterogeneous tumor cells. These components influence one another and thus, contribute to tumor progression and metastasis in either a positive or negative way. Therefore, a better understanding of the tumor microenvironment offers new insights for improving cancer therapies (79, 80). Cytokines are one of the key mediators for interactions between immune and nonimmune cells in the tumor microenvironment (TME) (81). It has been shown to have a different role that is isoform dependent since many cells express IL-32. However, it is not clear yet how IL-32 contributes to the different tumor types including stromal tumor microenvironment.

In a study investigating the IL-32 effect in the pathogenesis of endometriosis as an example of stromal cancer, IL-32 showed a correlation in cancer progression. This study revealed that the IL-32 concentration in the peritoneal fluid was drastically greater in patients of advanced-stage endometriosis as compared with the controls. Moreover, they showed that IL-32α and IL-32γ significantly increased cellular viability, proliferating cell nuclear antigen expression, and invasive ability (82).

Several studies showed that the overexpression of IL-32, specifically α, β, and γ were able to reduce tumor growth through inducing apoptosis in tumor cells, which led to CD8^+^ T-cell responses (15, 17, 33). Nevertheless, other than the antitumor effect, IL-32 demonstrates a monocyte differentiation stimulator as well as cytokine production. Moreover, it has been reported for its ability to activate T cells and therefore stimulate antigen presentation utilizing dendritic cells (DCs). On the contrary, functional studies demonstrated that IL-32γ induced PD-L1 expression on monocytes but not tumor cells, which may contribute to local immunosuppression and therefore are candidates for cotargeting in combination treatment regimens. IL-32γ expression correlates with a treatment-resistant dedifferentiated genetic signature and genes related to T-cell infiltration. This was reported in melanoma cells, suggesting it influences nonmelanoma cells in the tumor microenvironment, such as myeloid cells (83).

More recently, IL-32γ potentiates antitumor immunity in melanoma as the antitumor microenvironment. This result is shown to be enriched in mature DC and M1 macrophages resulting in enhancing the recurrence of activated tumor-specific CD8^+^ T cells to generate antitumor immunity. Therefore, IL-32 resulted in reducing tumor growth and rendering immune checkpoint blockade resistance (35). On the other hand, IL-32β stimulates the activation-induced apoptosis of T cells, NK cell cytotoxicity toward tumor cells like IL-32γ in the activation of monocyte differentiation. In addition, IL-32α is shown to be a stimulator of NK cell cytotoxicity, whereas IL-32θ has been shown as an inhibitory effect on monocyte differentiation and cytokine production (36, 84–88). However, better characterization of the tumor microenvironment is needed to understand how different cell types in the tumor microenvironment are influenced by IL-32.

Moreover, how IL-32 isoforms implicated each other is another key factor in overall response to cancer. As we mentioned above, the possibility of IL-32 in exhibiting an isoform switching and self-regulation between IL-32β and IL-32γ was reported (41). Likewise, isoform δ of IL-32 was found to modulate another isoform, IL-32β, by interacting with it and thus inhibiting its production of IL-10 (89). Both observations suggest that IL-32 performs its feedback regulation through its isoforms.

## Implications of IL-32 Polymorphisms in Cancer

Changes in the genetic material provide different effects within individuals and populations. Recently, several studies have demonstrated the impact of IL-32 polymorphisms on cancer progression. Moreover, IL-32 SNPs were studied and reviewed with their association to disease outcome (90–95), and by 2021, one review performed a meta-analysis to evaluate the SNPs in malignancy (96). Up to now, three polymorphisms of IL-32 were found to be associated with the progression of several cancers that are rs28372698, rs12934561, and rs2015620 (**Table 1**).

**Table 1:** IL-32 polymorphisms and their associated cancers.

IL-32 SNP	Chromosome location^a^	Type	Associated cancer/s	SNP interaction	Ref
**Rs28372698**	3,065,110	Noncoding/upstream variant	Thyroid carcinoma, lung, endometrial, ovarian, gastric cancer, bladder cancer, and colorectal cancer	rs4073 (IL-8)–gastric cancer	(97–103)
**Rs12934561**	3,068,864	Noncoding/lntron variant	Squamous carcinoma, and bladder cancer		(98, 102)
**Rs2015620**	3,063,897	Noncoding	Gastric cancer	rs917997 (IL-18RAP), rs1179251 (IL-22)	(103)

a Based on human genome build 38: GRCh38.

SNP rs28372698 was found in many cancers including thyroid carcinoma and lung, endometrial, ovarian, gastric, bladder, and colorectal cancers that are related to the higher expression of IL-32 resulting in cancer progression (96–104). In thyroid carcinoma, this polymorphism revealed higher expression of isoform IL−32γ that increased the risk of tumor development (104). In a study to evaluate cytokine polymorphisms and their association with gastric cancer, this SNP (rs28372698) of IL-32 has shown no association. However, when the patient has another SNP, IL-8 rs4073, there was an interaction between both SNPs and thus suggested increased gastric cancer risk (103).

Interestingly, another study on the Chinese population revealed that IL-32 SNP rs2015620 is highly associated with the risk of gastric cancer by interacting with two more SNPs, IL-18RAP rs917997 and IL-22 rs1179251 (101). However, these studies were subjected to two different populations, Chinese and Chilean; the reason why IL-32 SNP has a different effect. Although studies on IL-32 SNPs are not dispersed in the world, yet according to the published data, SNP rs28372698 showed high cancer influence on the Chinese population.

Moreover, this SNP was linked to colorectal cancer in the Swedish cohort but not reported in the Chinese colorectal cancer patients (99). Both IL-32 SNPs of rs28372698 and rs12934561 have been correlated with bladder cancer processes (102). However, only rs12934561 was related to poor survival status in squamous carcinoma (98). Overall, these association studies were subjected to some limitations due to the limited population and selected population. A large-scale study must include more than one kind of population and ethnicity to discover the role of IL-32 SNPs in cancers.

## Conclusion

It conflicts in targeting therapy for IL-32 in cancer because IL-32 roles remain unclear, thus there is no specific direction for IL-32 in cancer therapy. However, some isoforms showed an inhibitory effect that can be administered exogenously to stop or reverse cancer progression such as IL-32θ for cytokine-based immunotherapy. Moreover, it was found that patients with higher expression of IL-32 demonstrated more aggressive cancers. In these cases, IL-32 can be targeted precisely to stop its progression role. There is a great gap in this matter even after selecting the IL-32 isoform for cancer therapy. A lot more studies are needed before this knowledge can be used clinically. This difficulty regarding IL-32 was addressed in a recent review considering interleukins in improving cancer therapies (4). Again, this is due to IL-32 showing no clear effect on cancer which differs based on IL-32 isoforms, cancer type, and genetic background.

## Author Contributions

Conceptualization: SS, SL, YH, SK, TN, AT, JH, HJ, YL, SY, Y-GK, and SHK. Funding acquisition: HJ, SHK. Supervision: SHK. Writing—original draft: SS, SL, YH, and SHK. Writing—review and editing: AT and SHK. All authors listed have made a substantial, direct, and intellectual contribution to the work and approved it for publication.

## Funding

This paper was written as part of Konkuk University’s research support program for its faculty on sabbatical leave in 2022. This work was supported by the National Research Foundation of Korea (NRF-2021R1F1A1057397). This research was supported by the Main Research Program (E0210602-02) of the Korea Food Research Institute (KFRI) funded by the Ministry of Science and ICT. S-YK and Y-GK were supported by NRF-2021M3A9G1026605. SL was supported by NRF-2019R1I1A1A01057699.

## Conflict of Interest

The authors declare that the research was conducted in the absence of any commercial or financial relationships that could be construed as a potential conflict of interest.

## Publisher’s Note

All claims expressed in this article are solely those of the authors and do not necessarily represent those of their affiliated organizations, or those of the publisher, the editors and the reviewers. Any product that may be evaluated in this article, or claim that may be made by its manufacturer, is not guaranteed or endorsed by the publisher.

## References

[1] Kim SH Han SY Azam T Yoon DY Dinarello CA. Interleukin-32: A Cytokine and Inducer of TNFalpha. Immunity (2005) 22(1):131–42. doi: 10.1016/S1074-7613(04)00380-2 15664165

[2] Hong JT Son DJ Lee CK Yoon DY Lee DH Park MH. Interleukin 32, Inflammation and Cancer. Pharmacol Ther (2017) 174:127–37. doi: 10.1016/j.pharmthera.2017.02.025 28223235

[3] Sohn DH Nguyen TT Kim S Shim S Lee S Lee Y. Structural Characteristics of Seven IL-32 Variants. Immune Netw (2019) 19(2):e8. doi: 10.4110/in.2019.19.e8 31089435 PMC6494766

[4] Briukhovetska D Dorr J Endres S Libby P Dinarello CA Kobold S. Interleukins in Cancer: From Biology to Therapy. Nat Rev Cancer (2021) 21(8):481–99. doi: 10.1038/s41568-021-00363-z PMC8173513 34083781

[5] Chen LG Guo JQ Li DD. Correlation Between Dynamic Change of IL-32 Level and Disease Development in Acute Leukemia Patients. Zhongguo Shi Yan Xue Ye Xue Za Zhi (2017) 25(3):688–92. doi: 10.7534/j.issn.1009-2137.2017.03.010 28641619

[6] Li H Li J Cheng J Chen X Zhou L Li Z. AMLderived Mesenchymal Stem Cells Upregulate CTGF Expression Through the BMP Pathway and Induce K562ADM Fusiform Transformation and Chemoresistance. Oncol Rep (2019) 42(3):1035–46. doi: 10.3892/or.2019.7237 PMC6667869 31322275

[7] Zhai JM An YH Wang W Fan YG Yao GL. IL-32 Expression Indicates Unfavorable Prognosis in Patients With Colon Cancer. Oncol Lett (2019) 17(5):4655–60. doi: 10.3892/ol.2019.10136 PMC6447918 30988822

[8] Park JS Choi SY Lee JH Lee M Nam ES Jeong AL. Interleukin-32beta Stimulates Migration of MDA-MB-231 and MCF-7cells *via* the VEGF-STAT3 Signaling Pathway. Cell Oncol (Dordr) (2013) 36(6):493–503. doi: 10.1007/s13402-013-0154-4 24114327 PMC13007480

[9] Ternette N Olde Nordkamp MJM Muller J Anderson AP Nicastri A Hill AVS. Immunopeptidomic Profiling of HLA-A2-Positive Triple Negative Breast Cancer Identifies Potential Immunotherapy Target Antigens. Proteomics (2018) 18(12):e1700465. doi: 10.1002/pmic.201700465 29786170 PMC6032843

[10] Zeng Q Li S Zhou Y Ou W Cai X Zhang L. Interleukin-32 Contributes to Invasion and Metastasis of Primary Lung Adenocarcinoma *via* NF-kappaB Induced Matrix Metalloproteinases 2 and 9 Expression. Cytokine (2014) 65(1):24–32. doi: 10.1016/j.cyto.2013.09.017 24140068

[11] Takagi K Imura J Shimomura A Noguchi A Minamisaka T Tanaka S. Establishment of Highly Invasive Pancreatic Cancer Cell Lines and the Expression of IL-32. Oncol Lett (2020) 20(3):2888–96. doi: 10.3892/ol.2020.11825 PMC7400074 32782605

[12] Erturk K Tastekin D Serilmez M Bilgin E Bozbey HU Vatansever S. Clinical Significance of Serum Interleukin-29, Interleukin-32, and Tumor Necrosis Factor Alpha Levels in Patients With Gastric Cancer. Tumour Biol (2016) 37(1):405–12. doi: 10.1007/s13277-015-3829-9 26219901

[13] Kim MS Kang JW Jeon JS Kim JK Kim JW Hong J. IL-32theta Gene Expression in Acute Myeloid Leukemia Suppresses TNF-Alpha Production. Oncotarget (2015) 6(38):40747–61. doi: 10.18632/oncotarget.5688 PMC4747366 26516703

[14] Cheon S Lee JH Park S Bang SI Lee WJ Yoon DY. Overexpression of IL-32alpha Increases Natural Killer Cell-Mediated Killing Through Up-Regulation of Fas and UL16-Binding Protein 2 (ULBP2) Expression in Human Chronic Myeloid Leukemia Cells. J Biol Chem (2011) 286(14):12049–55. doi: 10.1074/jbc.M110.159756 PMC3069408 21321117

[15] Yun HM Oh JH Shim JH Ban JO Park KR Kim JH. Antitumor Activity of IL-32beta Through the Activation of Lymphocytes, and the Inactivation of NF-kappaB and STAT3 Signals. Cell Death Dis (2013) 4:e640. doi: 10.1038/cddis.2013.166 23703385 PMC3674373

[16] Park ES Yoo JM Yoo HS Yoon DY Yun YP Hong J. IL-32gamma Enhances TNF-Alpha-Induced Cell Death in Colon Cancer. Mol Carcinog (2014) 53 Suppl 1:E23–35. doi: 10.1002/mc.21990 23255489

[17] Yun HM Park KR Kim EC Han SB Yoon DY Hong JT. IL-32alpha Suppresses Colorectal Cancer Development *via* TNFR1-Mediated Death Signaling. Oncotarget (2015) 6(11):9061–72. doi: 10.18632/oncotarget.3197 PMC4496202 25909160

[18] Bak Y Kwon T Bak IS Hong J Yu DY Yoon DY. IL-32theta Inhibits Stemness and Epithelial-Mesenchymal Transition of Cancer Stem Cells *via* the STAT3 Pathway in Colon Cancer. Oncotarget (2016) 7(6):7307–17. doi: 10.18632/oncotarget.7007 PMC4872787 26824417

[19] Lee YS Kim KC Mongre RK Kim JY Kim YR Choi DY. IL-32gamma Suppresses Lung Cancer Stem Cell Growth via Inhibition of ITGAV-Mediated STAT5 Pathway. Cell Death Dis (2019) 10(7):506. doi: 10.1038/s41419-019-1737-4 31263095 PMC6602938

[20] Choi JD Bae SY Hong JW Azam T Dinarello CA Her E. Identification of the Most Active Interleukin-32 Isoform. Immunology (2009) 126(4):535–42. doi: 10.1111/j.1365-2567.2008.02917.x PMC2673365 18771438

[21] Kang JW Park YS Lee DH Kim MS Bak Y Ham SY. Interaction Network Mapping Among IL-32 Isoforms. Biochimie (2014) 101:248–51. doi: 10.1016/j.biochi.2014.01.013 24472437

[22] Han L Chen S Chen Z Zhou B Zheng Y Shen L. Interleukin 32 Promotes Foxp3(+) Treg Cell Development and CD8(+) T Cell Function in Human Esophageal Squamous Cell Carcinoma Microenvironment. Front Cell Dev Biol (2021) 9:704853. doi: 10.3389/fcell.2021.704853 34414188 PMC8369465

[23] Cui Y Sun Z Li X Leng C Zhang L Fu X. Expression and Clinical Significance of Cyclooxygenase-2 and Interleukin-32 in Primary Gastric B-Cell Lymphoma. Oncol Lett (2016) 11(1):693–8. doi: 10.3892/ol.2015.3950 PMC4727151 26870269

[24] Suga H Sugaya M Miyagaki T Kawaguchi M Fujita H Asano Y. The Role of IL-32 in Cutaneous T-Cell Lymphoma. J Invest Dermatol (2014) 134(5):1428–35. doi: 10.1038/jid.2013.488 24226419

[25] Lin X Yang L Wang G Zi F Yan H Guo X. Interleukin-32alpha Promotes the Proliferation of Multiple Myeloma Cells by Inducing Production of IL-6 in Bone Marrow Stromal Cells. Oncotarget (2017) 8(54):92841–54. doi: 10.18632/oncotarget.21611 PMC5696226 29190960

[26] Xia Y Cortez-Retamozo V Niederkofler V Salie R Chen S Samad TA. Dragon (Repulsive Guidance Molecule B) Inhibits IL-6 Expression in Macrophages. J Immunol (2011) 186(3):1369–76. doi: 10.4049/jimmunol.1002047 PMC3670585 21187450

[27] Anderson GJ Darshan D. Small-Molecule Dissection of BMP Signaling. Nat Chem Biol (2008) 4(1):15–6. doi: 10.1038/nchembio0108-15 18084273

[28] Eckhardt BL Cao Y Redfern AD Chi LH Burrows AD Roslan S. Activation of Canonical BMP4-SMAD7 Signaling Suppresses Breast Cancer Metastasis. Cancer Res (2020) 80(6):1304–15. doi: 10.1158/0008-5472.CAN-19-0743 31941699

[29] Leeman-Neill RJ Bhagat G. BCL6 as a Therapeutic Target for Lymphoma. Expert Opin Ther Targets (2018) 22(2):143–52. doi: 10.1080/14728222.2018.1420782 29262721

[30] Park YS Kang JW Lee DH Kim MS Bak Y Yang Y. Interleukin-32alpha Downregulates the Activity of the B-Cell CLL/lymphoma 6 Protein by Inhibiting Protein Kinase Cepsilon-Dependent SUMO-2 Modification. Oncotarget (2014) 5(18):8765–77. doi: 10.18632/oncotarget.2364 PMC4226720 25245533

[31] Aziz MH Manoharan HT Sand JM Verma AK. Protein Kinase Cepsilon Interacts With Stat3 and Regulates Its Activation That Is Essential for the Development of Skin Cancer. Mol Carcinog (2007) 46(8):646–53. doi: 10.1002/mc.20356 17583567

[32] Gorin MA Pan Q. Protein Kinase C Epsilon: An Oncogene and Emerging Tumor Biomarker. Mol Cancer (2009) 8:9. doi: 10.1186/1476-4598-8-9 19228372 PMC2647895

[33] Oh JH Cho MC Kim JH Lee SY Kim HJ Park ES. IL-32gamma Inhibits Cancer Cell Growth Through Inactivation of NF-kappaB and STAT3 Signals. Oncogene (2011) 30(30):3345–59. doi: 10.1038/onc.2011.52 PMC3145890 21423208

[34] Park MH Song MJ Cho MC Moon DC Yoon DY Han SB. Interleukin-32 Enhances Cytotoxic Effect of Natural Killer Cells to Cancer Cells *via* Activation of Death Receptor 3. Immunology (2012) 135(1):63–72. doi: 10.1111/j.1365-2567.2011.03513.x 22043900 PMC3246653

[35] Gruber T Kremenovic M Sadozai H Rombini N Baeriswyl L Maibach F. IL-32gamma Potentiates Tumor Immunity in Melanoma. JCI Insight (2020) 5(18). doi: 10.1172/jci.insight.138772 PMC7526542 32841222

[36] Khawar MB Mukhtar M Abbasi MH Sheikh N. IL-32theta: A Recently Identified Anti-Inflammatory Variant of IL-32 and Its Preventive Role in Various Disorders and Tumor Suppressor Activity. Am J Transl Res (2017) 9(11):4726–37. PMC5714761 29218075

[37] Diakowska D Krzystek-Korpacka M. Local and Systemic Interleukin-32 in Esophageal, Gastric, and Colorectal Cancers: Clinical and Diagnostic Significance. Diagnostics (Basel) (2020) 10(10):785. doi: 10.3390/diagnostics10100785 PMC7600995 33020452

[38] Yang Y Wang Z Zhou Y Wang X Xiang J Chen Z. Dysregulation of Over-Expressed IL-32 in Colorectal Cancer Induces Metastasis. World J Surg Oncol (2015) 13:146. doi: 10.1186/s12957-015-0552-3 25889282 PMC4414001

[39] Catalan V Gomez-Ambrosi J Rodriguez A Ramirez B Ortega VA Hernandez-Lizoain JL. IL-32alpha-Induced Inflammation Constitutes a Link Between Obesity and Colon Cancer. Oncoimmunology (2017) 6(7):e1328338. doi: 10.1080/2162402X.2017.1328338 28811968 PMC5543901

[40] Park JS Lee S Jeong AL Han S Ka HI Lim JS. Hypoxia-Induced IL-32beta Increases Glycolysis in Breast Cancer Cells. Cancer Lett (2015) 356(2 Pt B):800–8. doi: 10.1016/j.canlet.2014.10.030 25449783

[41] Heinhuis B Koenders MI van de Loo FA Netea MG van den Berg WB Joosten LA. Inflammation-Dependent Secretion and Splicing of IL-32{Gamma} in Rheumatoid Arthritis. Proc Natl Acad Sci USA (2011) 108(12):4962–7. doi: 10.1073/pnas.1016005108 PMC3064318 21383200

[42] Wang S Chen F Tang L. IL-32 Promotes Breast Cancer Cell Growth and Invasiveness. Oncol Lett (2015) 9(1):305–7. doi: 10.3892/ol.2014.2641 PMC4246643 25435980

[43] Pham TH Park HM Kim J Hong JT Yoon DY. Interleukin-32theta Triggers Cellular Senescence and Reduces Sensitivity to Doxorubicin-Mediated Cytotoxicity in MDA-MB-231 Cells. Int J Mol Sci (2021) 22(9). doi: 10.3390/ijms22094974 PMC8124300 34067074

[44] Pham TH Bak Y Kwon T Kwon SB Oh JW Park JH. Interleukin-32theta Inhibits Tumor-Promoting Effects of Macrophage-Secreted CCL18 in Breast Cancer. Cell Commun Signal (2019) 17(1):53. doi: 10.1186/s12964-019-0374-y 31126309 PMC6534939

[45] Reyland ME. Protein Kinase Cdelta and Apoptosis. Biochem Soc Trans (2007) 35(Pt 5):1001–4. doi: 10.1042/BST0351001 17956263

[46] Kang YH Park MY Yoon DY Han SR Lee CI Ji NY. Dysregulation of Overexpressed IL-32alpha in Hepatocellular Carcinoma Suppresses Cell Growth and Induces Apoptosis Through Inactivation of NF-kappaB and Bcl-2. Cancer Lett (2012) 318(2):226–33. doi: 10.1016/j.canlet.2011.12.023 22198481

[47] Zhao WB Wang QL Xu YT Xu SF Qiu Y Zhu F. Overexpression of Interleukin-32alpha Promotes Invasion by Modulating VEGF in Hepatocellular Carcinoma. Oncol Rep (2018) 39(3):1155–62. doi: 10.3892/or.2017.6162 29286122

[48] Iliaz R Akyuz U Tekin D Serilmez M Evirgen S Cavus B. Role of Several Cytokines and Adhesion Molecules in the Diagnosis and Prediction of Survival of Hepatocellular Carcinoma. Arab J Gastroenterol (2016) 17(4):164–7. doi: 10.1016/j.ajg.2016.10.002 27916547

[49] Nishida A Andoh A Inatomi O Fujiyama Y. Interleukin-32 Expression in the Pancreas. J Biol Chem (2009) 284(26):17868–76. doi: 10.1074/jbc.M900368200 PMC2719425 19386602

[50] Tsai CY Wang CS Tsai MM Chi HC Cheng WL Tseng YH. Interleukin-32 Increases Human Gastric Cancer Cell Invasion Associated With Tumor Progression and Metastasis. Clin Cancer Res (2014) 20(9):2276–88. doi: 10.1158/1078-0432.CCR-13-1221 24602839

[51] Ishigami S Arigami T Uchikado Y Setoyama T Kita Y Sasaki K. IL-32 Expression Is an Independent Prognostic Marker for Gastric Cancer. Med Oncol (2013) 30(2):472. doi: 10.1007/s12032-013-0472-4 23479179

[52] Sakitani K Hirata Y Hayakawa Y Serizawa T Nakata W Takahashi R. Role of Interleukin-32 in Helicobacter Pylori-Induced Gastric Inflammation. Infect Immun (2012) 80(11):3795–803. doi: 10.1128/IAI.00637-12 PMC3486038 22890997

[53] Seo EH Kang J Kim KH Cho MC Lee S Kim HJ. Detection of Expressed IL-32 in Human Stomach Cancer Using ELISA and Immunostaining. J Microbiol Biotechnol (2008) 18(9):1606–12. 18852519

[54] Chang WJ Du Y Zhao X Ma LY Cao GW. Inflammation-Related Factors Predicting Prognosis of Gastric Cancer. World J Gastroenterol (2014) 20(16):4586–96. doi: 10.3748/wjg.v20.i16.4586 PMC4000495 24782611

[55] Yousif NG Al-Amran FG Hadi N Lee J Adrienne J. Expression of IL-32 Modulates NF-kappaB and P38 MAP Kinase Pathways in Human Esophageal Cancer. Cytokine (2013) 61(1):223–7. doi: 10.1016/j.cyto.2012.09.022 23107826

[56] O'Sullivan KE Phelan JJ O'Hanlon C Lysaght J O'Sullivan JN Reynolds JV. The Role of Inflammation in Cancer of the Esophagus. Expert Rev Gastroenterol Hepatol (2014) 8(7):749–60. doi: 10.1586/17474124.2014.913478 24857183

[57] Simon D Radonjic-Hosli S Straumann A Yousefi S Simon HU. Active Eosinophilic Esophagitis Is Characterized by Epithelial Barrier Defects and Eosinophil Extracellular Trap Formation. Allergy (2015) 70(4):443–52. doi: 10.1111/all.12570 25620273

[58] Nold-Petry CA Rudloff I Baumer Y Ruvo M Marasco D Botti P. IL-32 Promotes Angiogenesis. J Immunol (2014) 192(2):589–602. doi: 10.4049/jimmunol.1202802 24337385 PMC4007307

[59] Varner JA Cheresh DA. Integrins and Cancer. Curr Opin Cell Biol (1996) 8(5):724–30. doi: 10.1016/S0955-0674(96)80115-3 8939661

[60] Liu Z Wang F Chen X. Integrin Alpha(V)Beta(3)-Targeted Cancer Therapy. Drug Dev Res (2008) 69(6):329–39. doi: 10.1002/ddr.20265 PMC2901818 20628538

[61] Grunstein J Roberts WG Mathieu-Costello O Hanahan D Johnson RS. Tumor-Derived Expression of Vascular Endothelial Growth Factor Is a Critical Factor in Tumor Expansion and Vascular Function. Cancer Res (1999) 59(7):1592–8. 10197634

[62] Waugh DJ Wilson C. The Interleukin-8 Pathway in Cancer. Clin Cancer Res (2008) 14(21):6735–41. doi: 10.1158/1078-0432.CCR-07-4843 18980965

[63] Kessenbrock K Plaks V Werb Z. Matrix Metalloproteinases: Regulators of the Tumor Microenvironment. Cell (2010) 141(1):52–67. doi: 10.1016/j.cell.2010.03.015 20371345 PMC2862057

[64] Gialeli C Theocharis AD Karamanos NK. Roles of Matrix Metalloproteinases in Cancer Progression and Their Pharmacological Targeting. FEBS J (2011) 278(1):16–27. doi: 10.1111/j.1742-4658.2010.07919.x 21087457

[65] Abdel-Hamid NM Abass SA. Matrix Metalloproteinase Contribution in Management of Cancer Proliferation, Metastasis and Drug Targeting. Mol Biol Rep (2021) 48(9):6525–38. doi: 10.1007/s11033-021-06635-z 34379286

[66] Sorrentino C Di Carlo E. Expression of IL-32 in Human Lung Cancer is Related to the Histotype and Metastatic Phenotype. Am J Respir Crit Care Med (2009) 180(8):769–79. doi: 10.1164/rccm.200903-0400OC 19628777

[67] Zhou Y Hu Z Li N Jiang R. Interleukin-32 Stimulates Osteosarcoma Cell Invasion and Motility *via* AKT Pathway-Mediated MMP-13 Expression. Int J Mol Med (2015) 35(6):1729–33. doi: 10.3892/ijmm.2015.2159 25846944

[68] Sun JH Fan N Zhang Y. Correlation Between Serum Level of Chemokine (C-C Motif) Ligand 18 and Poor Prognosis in Breast Cancer. Genet Mol Res (2016) 15(3):gmr.15038632. doi: 10.4238/gmr.15038632 27706714

[69] Huang H Li J Hu WJ Chen C Luo HQ Tang XD. The Serum Level of CC Chemokine Ligand 18 Correlates With the Prognosis of Non-Small Cell Lung Cancer. Int J Biol Markers (2019) 34(2):156–62. doi: 10.1177/1724600819829758 31046524

[70] Meng F Li W Li C Gao Z Guo K Song S. CCL18 Promotes Epithelial-Mesenchymal Transition, Invasion and Migration of Pancreatic Cancer Cells in Pancreatic Ductal Adenocarcinoma. Int J Oncol (2015) 46(3):1109–20. doi: 10.3892/ijo.2014.2794 25502147

[71] Darnell JE Jr. STATs and Gene Regulation. Science (1997) 277(5332):1630–5. doi: 10.1126/science.277.5332.1630 9287210

[72] Huang S. Regulation of Metastases by Signal Transducer and Activator of Transcription 3 Signaling Pathway: Clinical Implications. Clin Cancer Res (2007) 13(5):1362–6. doi: 10.1158/1078-0432.CCR-06-2313 17332277

[73] Kamran MZ Patil P Gude RP. Role of STAT3 in Cancer Metastasis and Translational Advances. BioMed Res Int (2013) 2013 p:421821. doi: 10.1155/2013/421821 24199193 PMC3807846

[74] Xie TX Wei D Liu M Gao AC Ali-Osman F Sawaya R. Stat3 Activation Regulates the Expression of Matrix Metalloproteinase-2 and Tumor Invasion and Metastasis. Oncogene (2004) 23(20):3550–60. doi: 10.1038/sj.onc.1207383 15116091

[75] Dechow TN Pedranzini L Leitch A Leslie K Gerald WL Linkov I. Requirement of Matrix Metalloproteinase-9 for the Transformation of Human Mammary Epithelial Cells by Stat3-C. Proc Natl Acad Sci USA (2004) 101(29):10602–7. doi: 10.1073/pnas.0404100101 PMC489981 15249664

[76] Chen Z Han ZC. STAT3: A Critical Transcription Activator in Angiogenesis. Med Res Rev (2008) 28(2):185–200. doi: 10.1002/med.20101 17457812

[77] Yahata Y Shirakata Y Tokumaru S Yamasaki K Sayama K Hanakawa Y. Nuclear Translocation of Phosphorylated STAT3 Is Essential for Vascular Endothelial Growth Factor-Induced Human Dermal Microvascular Endothelial Cell Migration and Tube Formation. J Biol Chem (2003) 278(41):40026–31. doi: 10.1074/jbc.M301866200 12874294

[78] Oh MK Park HJ Kim NH Park SJ Park IY Kim IS. Hypoxia-Inducible Factor-1alpha Enhances Haptoglobin Gene Expression by Improving Binding of STAT3 to the Promoter. J Biol Chem (2011) 286(11):8857–65. doi: 10.1074/jbc.M110.150557 PMC3058976 21224490

[79] Arneth B. Tumor Microenvironment. Medicina (Kaunas) (2019) 56(1):15. doi: 10.3390/medicina56010015 PMC7023392 31906017

[80] Wu T Dai Y. Tumor Microenvironment and Therapeutic Response. Cancer Lett (2017) 387:61–8. doi: 10.1016/j.canlet.2016.01.043 26845449

[81] Hinshaw DC Shevde LA. The Tumor Microenvironment Innately Modulates Cancer Progression. Cancer Res (2019) 79(18):4557–66. doi: 10.1158/0008-5472.CAN-18-3962 PMC6744958 31350295

[82] Lee MY Kim SH Oh YS Heo SH Kim KH Chae HD. Role of Interleukin-32 in the Pathogenesis of Endometriosis: *In Vitro*, Human and Transgenic Mouse Data. Hum Reprod (2018) 33(5):807–16. doi: 10.1093/humrep/dey055 29562285

[83] Taube JM Young GD McMiller TL Chen S Salas JT Pritchard TS. Differential Expression of Immune-Regulatory Genes Associated With PD-L1 Display in Melanoma: Implications for PD-1 Pathway Blockade. Clin Cancer Res (2015) 21(17):3969–76. doi: 10.1158/1078-0432.CCR-15-0244 PMC4558237 25944800

[84] Kim YG Lee CK Oh JS Kim SH Kim KA Yoo B. Effect of Interleukin-32gamma on Differentiation of Osteoclasts From CD14+ Monocytes. Arthritis Rheum (2010) 62(2):515–23. doi: 10.1002/art.27197 20112365

[85] Netea MG Lewis EC Azam T Joosten LA Jaekal J Bae SY. Interleukin-32 Induces the Differentiation of Monocytes Into Macrophage-Like Cells. Proc Natl Acad Sci USA (2008) 105(9):3515–20. doi: 10.1073/pnas.0712381105 PMC2265135 18296636

[86] Jeong HJ Nam SY Oh HA Han NR Kim YS Moon PD. Interleukin-32-Induced Thymic Stromal Lymphopoietin Plays a Critical Role in Macrophage Differentiation Through the Activation of Caspase-1 In Vitro. Arthritis Res Ther (2012) 14(6):R259. doi: 10.1186/ar4104 23190696 PMC3674606

[87] Marcondes AM Mhyre AJ Stirewalt DL Kim SH Dinarello CA Deeg HJ. Dysregulation of IL-32 in Myelodysplastic Syndrome and Chronic Myelomonocytic Leukemia Modulates Apoptosis and Impairs NK Function. Proc Natl Acad Sci USA (2008) 105(8):2865–70. doi: 10.1073/pnas.0712391105 PMC2268551 18287021

[88] Goda C Kanaji T Kanaji S Tanaka G Arima K Ohno S. Involvement of IL-32 in Activation-Induced Cell Death in T Cells. Int Immunol (2006) 18(2):233–40. doi: 10.1093/intimm/dxh339 16410314

[89] Kang JW Park YS Lee DH Kim MS Bak Y Park SH. Interleukin-32delta Interacts With IL-32beta and Inhibits IL-32beta-Mediated IL-10 Production. FEBS Lett (2013) 587(23):3776–81. doi: 10.1016/j.febslet.2013.10.019 24396867

[90] Morsaljahan Z Rafiei A Valadan R Abedini M Pakseresht M Khajavi R. Association Between Interleukin-32 Polymorphism and Multiple Sclerosis. J Neurol Sci (2017) 379:144–50. doi: 10.1016/j.jns.2017.05.045 28716229

[91] Zheng S Li M Pan X Huang X. Association of IL-32 Rs28372698 Polymorphism With Active Chronic HBV Infection. J Med Virol (2021) 93(11):6236–40. doi: 10.1002/jmv.27140 34138488

[92] Mazlum F Gharesi-Fard B Hadinedoushan H Bakhshizadeh Ghashti Y. Association Between Interleukin-32 Gene Polymorphism and Susceptibility to Preeclampsia. Hypertens Pregnancy (2021) 40(3):218–25. doi: 10.1080/10641955.2021.1958836 34346819

[93] Damen MS Agca R Holewijn S de Graaf J Dos Santos JC van Riel PL. IL-32 Promoter SNP Rs4786370 Predisposes to Modified Lipoprotein Profiles in Patients With Rheumatoid Arthritis. Sci Rep (2017) 7:41629. doi: 10.1038/srep41629 28134327 PMC5278556

[94] Zhang M Xu WD Zhu Y Wen PF Leng RX Pan HF. Serum Levels of Cytokines in Systemic Lupus Erythematosus: Association Study in a Chinese Population. Z Rheumatol (2014) 73(3):277–80. doi: 10.1007/s00393-013-1274-y 24310228

[95] Alehagen U Shamoun L Dimberg JI Wagsater D. Increased Mortality in the a/A Genotype of the SNP Rs28372698 of Interleukin 32. Exp Ther Med (2021) 21(2):127. doi: 10.3892/etm.2020.9559 33376509 PMC7751449

[96] Jafrin S Abdul Aziz M Islam MS. Impact of Interleukin-32 Germ-Line Rs28372698 and Intronic Rs12934561 Polymorphisms on Cancer Development: A Systematic Review and Meta-Analysis. Int Immunopharmacol (2021) 99:107964. doi: 10.1016/j.intimp.2021.107964 34271417

[97] Yu X Zhou B Zhang Z Gao Q Wang Y Song Y. Significant Association Between IL-32 Gene Polymorphisms and Susceptibility to Endometrial Cancer in Chinese Han Women. Tumour Biol (2015) 36(7):5265–72. doi: 10.1007/s13277-015-3186-8 25663496

[98] Wang Y Yang Y Zhu Y Li L Chen F Zhang L. Polymorphisms and Expression of IL-32: Impact on Genetic Susceptibility and Clinical Outcome of Lung Cancer. Biomarkers (2017) 22(2):165–70. doi: 10.1080/1354750X.2016.1252956 27775437

[99] Shamoun L Kolodziej B Andersson RE Dimberg J. Protein Expression and Genetic Variation of IL32 and Association With Colorectal Cancer in Swedish Patients. Anticancer Res (2018) 38(1):321–8. doi: 10.21873/anticanres.12225 29277790

[100] Luo X Bai P Li Q Zhang Y Song Y Su M. Association Between Interleukin-32 Polymorphisms and Ovarian Cancer in the Chinese Han Population. Int J Clin Exp Pathol (2020) 13(7):1733–8. PMC7414470 32782697

[101] Wang YM Li ZX Tang FB Zhang Y Zhou T Zhang L. Association of Genetic Polymorphisms of Interleukins With Gastric Cancer and Precancerous Gastric Lesions in a High-Risk Chinese Population. Tumour Biol (2016) 37(2):2233–42. doi: 10.1007/s13277-015-4022-x 26358252

[102] Yang J Jian Z Shen P Bai Y Tang Y Wang J. Associations Between Interleukin-32 Gene Polymorphisms Rs12934561 and Rs28372698 and Susceptibilities to Bladder Cancer and the Prognosis in Chinese Han Population. Dis Markers (2020) 2020:8860445. doi: 10.1155/2020/8860445 33204366 PMC7661138

[103] Gonzalez-Hormazabal P Musleh M Bustamante M Stambuk J Escandar S Valladares H. Role of Cytokine Gene Polymorphisms in Gastric Cancer Risk in Chile. Anticancer Res (2014) 34(7):3523–30. 24982364

[104] Plantinga TS Costantini B Heinhuis A Huijbers G Semango B Kusters. A Promoter Polymorphism in Human Interleukin-32 Modulates its Expression and Influences the Risk and the Outcome of Epithelial Cell-Derived Thyroid Carcinoma. Carcinogenesis (2013) 34(7):1529–35. doi: 10.1093/carcin/bgt092 23486016

